# An Optimization-Based Initial Alignment and Calibration Algorithm of Land-Vehicle SINS In-Motion

**DOI:** 10.3390/s18072081

**Published:** 2018-06-28

**Authors:** Kang Gao, Shunqing Ren, Xijun Chen, Zhenhuan Wang

**Affiliations:** Space Control and Inertial Technology Research Center, Harbin Institute of Technology, Harbin 150080, China; renshunqing@hit.edu.cn (S.R.); chenxijun@hit.edu.cn (X.C.); zhenhuanwang@gmail.com (Z.W.)

**Keywords:** strapdown inertial navigation system (SINS), initial alignment, odometer, optimized estimate, extended Kalman filter (EKF)

## Abstract

For a running freely land-vehicle strapdown inertial navigation system (SINS), the problems of self-calibration and attitude alignment need to be solved simultaneously. This paper proposes a complete alignment algorithm for the land vehicle navigation using Inertial Measurement Units (IMUs) and an odometer. A self-calibration algorithm is proposed based on the global observability analysis to calibrate the odometer scale factor and IMU misalignment angle, and the initial alignment and calibration method based on optimal algorithm is established to estimate the attitude and other system parameters. This new algorithm has the capability of self-initialization and calibration without any prior attitude and sensor noise information. Computer simulation results show that the performance of the proposed algorithm is superior to the extended Kalman filter (EKF) method during the oscillating attitude motions, and the vehicle test validates its advantages.

## 1. Introduction

The strapdown inertial navigation system (SINS) is an autonomous navigation system that uses inertial measurement units (IMUs) and initial navigation information to determine the attitude, position and velocity [1,2]. SINS is widely used in aviation, marine, land vehicle navigation and positioning because of its advantage of complete autonomy [3,4]. The SINS capability depends on the accuracy and rapidity of initial alignment process which is one of the key technologies in SINS [5,6]. The research about static base initial alignment has been done very well [7,8], however, how to complete the initial alignment of the inertial navigation system on moving base becomes an urgent problem to be solved.

Unlike the alignment on a static base, the alignment on a moving base usually requires the carrier motion information provided by some external device, for example, global positioning system (GPS), cameras, odometers and Doppler laser radars [9,10,11]. SINS/GPS integrated navigation is a commonly used integrated navigation mode [12,13,14]. GPS signals are vulnerable to interference or shielding, and the poor adaptability of GPS-assisted initial alignment system limits its application in the military field. Cameras are also a promising choice despite their tight dependence on easily-identified features with known positions on the path. Odometers are a kind of cost-effective and conveniently-deployed sensor for land vehicles, and odometer aided in-motion alignment is widely used because of its fully self-contained characteristics [15].

The Kalman filter is widely used in initial alignment [16,17]. In [18,19], Kalman filter-based initial alignment for SINS/Doppler velocity log (DVL) integration is studied. However, the Kalman filter requires knowledge of the noise statistics and a roughly known initial attitude that is hardly achieved when the vehicle running freely. If not properly initiated, DVL aided SINS initial alignment based on Kalman filtering would fail. 

By way of observability analysis, Wu systematically proposed a versatile strategy for self-contained land vehicle navigation using an IMU and an odometer [20,21]. In these papers, the INS attitude alignment is transformed into a “continuous” attitude determination problem using infinite vector observations, but the initial alignment is still implemented by the Kalman filter other than the optimal estimate algorithm. The coarse alignment algorithm based on optimal estimation for odometer aided SINS is studied in [22,23], in which the integration form of the velocity update equation in the body frame is used to give a rough initial attitude.

The optimization-based alignment (OBA) method with the aid of external velocity and position information provided by Global Navigation Satellite System (GNSS) is proposed in [24,25,26]. The OBA algorithm obtains an optimal attitude matrix through the q method to reduce random errors of attitude angles. However, the algorithms are not suitable for the IMU/odometer system because of the information provided by an odometer is different from the GNSS. In this paper, an optimization-based initial alignment and calibration algorithm of INS/odometer system is proposed, in which the attitude and the associated parameters including the odometer scale factor, lever arm, IMU misalignment angle and inertial sensor biases are estimated. The numerical and vehicle test results show that the performance of the proposed algorithm is superior to the extended Kalman filter (EKF) method during the angular motion.

The contents are organized as follows: Section 2 presents the frame definition and the SINS/odometer system model and conducts the observability analysis of system states. In Section 3, the numerical integration algorithm is derived and the joint estimation problem is posed as a unit quaternion-constrained optimization. Section 4 reports simulation and experiment results of the algorithm. Conclusions are finally drawn in Section 5.

## 2. Formulate Problem

### 2.1. System Description

**Figure 1:**
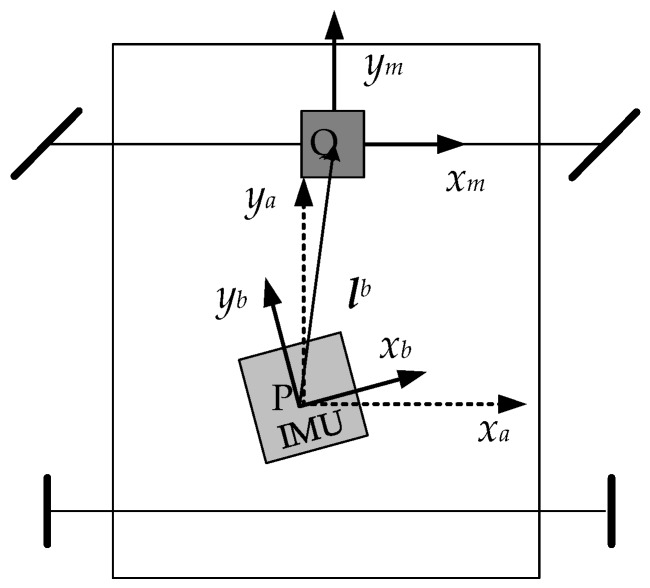
The relationship among a-frame, b-frame and m-frame. The IMU locate at the centroid of vehicle, the point P, and the odometer at the center of the front axle, the point Q.

In order to better understand and deduce the initial alignment and calibration algorithm, it is necessary to explain the related coordinate systems, that is, the Earth frame (e-frame), the navigation frame (n-frame), the vehicle frame (a-frame), the SINS frame (b-frame), and the odometer frame (m-frame). The relationship among a-frame, b-frame and m-frame is shown in Figure 1. These frames are defined in detail as follows.

e-frame: It is a frame fixed to the Earth and the origin is at its center; the *z_e_* axis goes along Earth polar axis pointing to the North Pole; the *x_e_* axis points to the intersection of the prime meridian and the equator; the *y_e_* axis and the *x_e_*, *z_e_* axis form a right-hand coordinate frame. The e-frame rotates around the Earth’s rotation with angular rate ω*_ie_*. 

n-frame: The local geographic frame (east-north-up, ENU) is selected as the navigation frame. Its origin is the centroid of the vehicle; the *z_n_* axis goes upward along the local geodetic vertical; the *y_n_* axis and *x_n_* axis horizontal north and east respectively.

a-frame: Its origin is the centroid of the vehicle, the point P as shown in Figure 1; the *x*_a_ axis shifts rightward along the vehicle’s transverse axis, the *y*_a_ axis forward along the longitudinal axis; the *z*_a_ axis upward.

b-frame: Its origin coincides with the origin of the vehicle frame; the axes aligned with the directions of the configuration of the three gyroscopes/accelerometers, which misalign the a-frame axes in attitude.

m-frame: Its origin is the center of the front axle, the point Q as shown in Figure 1; its coordinate axes are parallel to the three coordinate axes of b-frame; it is translated ***l****^b^* from the b-frame.

The navigation (attitude, velocity and position) rate equations in the reference n-frame are well known as [27]:

(1) $${\dot{C}}_{b}^{n}=C_{b}^{n}\left(\omega_{nb}^{b}\times \right),\;\omega_{nb}^{b}=\omega_{ib}^{b}-b_{g}-C_{n}^{b}\left(\omega_{ie}^{n}+\omega_{en}^{n}\right),$$

(2) $${\dot{v}}^{n}=C_{b}^{n}\left(f^{b}-b_{a}\right)-\left(2\omega_{ie}^{n}+\omega_{en}^{n}\right)\times v^{n}+g^{n},$$

where $C_{b}^{n}$ denotes the attitude matrix from b-frame to n-frame, $\omega_{nb}^{b}$ the body angular rate with respect to n-frame, $\omega_{ib}^{b}$ the body angular rate measured by gyroscopes in b-frame, $b_{g}$ the gyroscope bias, $\omega_{ie}^{n}$ denotes the e-frame rotation rate with respect to the inertial frame, $\omega_{en}^{n}$ the angular rate of the e-frame with respect to n-frame, $v^{n}$ the velocity relative to n-frame measured by SINS, $f^{b}$ the specific force measured by accelerometers in b-frame, $b_{a}$ the accelerometer bias, and $g^{n}$ the gravity vector. The 3 × 3 skew symmetric matrix (.×) is defined so that the cross product satisfies a×b=(a×)b for arbitrary two vectors. The gyroscope bias $b_{g}$ and the accelerometer bias $b_{a}$ are taken as random constants, i.e., ${\dot{b}}_{g}=0$, ${\dot{b}}_{a}=0$. All the quantities herein are functions of time and, if not stated, their time dependences are omitted for brevity.

Denote the IMU misalignment angle as $\alpha ={\left[\begin{array}{ccc} \alpha_{x} & \alpha_{y} & \alpha_{z} \end{array}\right]}^{T}$, that the misalignment angle between b-frame and m-frame, then the installation error matrix $C_{b}^{m}$ can be expressed as [27]:

(3) $$C_{b}^{m}=\left[\begin{array}{ccc} \cos\alpha_{y}\cos\alpha_{z}-\sin\alpha_{y}\sin\alpha_{x}\sin\alpha_{z} & \cos\alpha_{y}\sin\alpha_{z}+\sin\alpha_{y}\sin\alpha_{x}\cos\alpha_{z} & -\sin\alpha_{y}\cos\alpha_{x}\\ -\cos\alpha_{x}\sin\alpha_{z} & \cos\alpha_{x}\cos\alpha_{z} & \sin\alpha_{x}\\ \sin\alpha_{y}\cos\alpha_{z}+\cos\alpha_{y}\sin\alpha_{x}\sin\alpha_{z} & \sin\alpha_{y}\sin\alpha_{z}-\cos\alpha_{y}\sin\alpha_{x}\cos\alpha_{z} & \cos\alpha_{y}\cos\alpha_{x} \end{array}\right].$$

The misalignment angle is considered as constant, i.e., ${\dot{\alpha }}_{x}={\dot{\alpha }}_{y}={\dot{\alpha }}_{z}=0$.

Taking the odometer scale factor *k* and the lever arm $l^{b}$ into account, the speed at the odometer measurement point can be expressed as:

(4) $$y_{od}=k\cdot e_{2}{}^{T}C_{b}^{m}\left(C_{n}^{b}v_{s}^{n}+\omega_{nb}^{b}\times l^{b}\right),$$

where, $v_{s}^{n}$ is the velocity of the IMU measurement point expressed in n-frame, and $\omega_{nb}^{b}$ is the body angular rate with respect to the navigation frame, expressed in the b-frame. The odometer scale factor *k* and the lever arm $l^{b}$ are considered as constants, i.e., $\dot{k}=0,{\dot{l}}^{b}=0$.

For land-vehicle, the velocity in the plane perpendicular to the moving direction is assumed as zero, which is regarded as “virtual measurements”, i.e.:

(5) $$y_{nc}=\left[\begin{array}{l} 0\\ 0 \end{array}\right].$$

Merging Equations (4) and (5), the measurement equation is obtained as:

(6) $$y=diag\left\{\left[\begin{array}{ccc} 1 & k & 1 \end{array}\right]\right\}C_{b}^{m}\left(C_{n}^{b}v_{s}^{n}+\omega_{nb}^{b}\times l^{b}\right).$$

### 2.2. The System Observability Analysis

A system is said to be observable if the initial state could be derived from the known measurement and input information in finite time [28]. The observable state can be estimated by designed observer. In this section, authors investigate the observability of some system states directly from the basic observability concept.

Substituting Equation (3) into (6):

(7) $$C_{m}^{b}\left[\begin{array}{c} 0\\ y_{od}/k\\ 0 \end{array}\right]=\frac{y_{od}}{k}\left[\begin{array}{c} -\cos\alpha_{x}\sin\alpha_{z}\\ \cos\alpha_{x}\cos\alpha_{z}\\ \sin\alpha_{x} \end{array}\right]=C_{n}^{b}v_{s}^{n}+\omega_{nb}^{b}\times l^{b}.$$

It is obviously that the roll angle $\alpha_{y}$ has no effect on $y_{od}$, and it is unobservable. 

We rewrite Equation (2) as:

(8) $${\dot{v}}^{n}+\left(2\omega_{ie}^{n}+\omega_{en}^{n}\right)\times v^{n}-C_{b}^{n}\left(f^{b}-b_{a}\right)=g^{n}.$$

The time derivative of $v^{n}=C_{b}^{n}v^{b}$ is:

(9) $${\dot{v}}^{n}=C_{b}^{n}\left({\dot{v}}^{b}+\omega_{nb}^{b}\times v^{b}\right).$$

Substituting Equation (9) into Equation (8):

(10) $$C_{b}^{n}\left({\dot{v}}^{b}+\left(\omega_{ib}^{b}-b_{g}+\omega_{ie}^{b}\right)\times v^{b}-f^{b}+b_{a}\right)=g^{n}.$$

Rewrite Equation (6) as:

(11) $$v^{b}=C_{m}^{b}diag{\left\{\left[\begin{array}{ccc} 1 & k & 1 \end{array}\right]\right\}}^{-1}y-\omega_{nb}^{b}\times l^{b}.$$

Note $K=diag{\left\{\left[\begin{array}{ccc} 1 & k & 1 \end{array}\right]\right\}}^{-1}$, then we have

(12) $$v^{b}=C_{m}^{b}\mathrm{Ky}-\omega_{nb}^{b}\times l^{b}.$$

The derivative on both sides of Equation (12) is

(13) $${\dot{v}}^{b}=C_{m}^{b}K\dot{y}-{\dot{\omega }}_{nb}^{b}\times l^{b}.$$

Substituting Equation (13) into Equation (10), we have

(14) $$C_{b}^{n}\left[C_{m}^{b}K\dot{y}-{\dot{\omega }}_{nb}^{b}\times l^{b}+\left(\omega_{ib}^{b}-b_{g}+\omega_{ie}^{b}\right)\times \left(C_{m}^{b}Ky-{\dot{\omega }}_{nb}^{b}\times l^{b}\right)-f^{b}+b_{a}\right]=g^{n}.$$

Rewrite Equation (14) as

(15) $$\left[C_{m}^{b}K\dot{y}+\left(\omega_{ib}^{b}-b_{g}+\omega_{ie}^{b}\right)\times C_{m}^{b}Ky\right]-\left[{\dot{\omega }}_{nb}^{b}+\left(\omega_{ib}^{b}-b_{g}+\omega_{ie}^{b}\right)\times \omega_{nb}^{b}\right]\times l^{b}=C_{n}^{b}g^{n}+f^{b}-b_{a}.$$

If the carrier has no attitude maneuver, then $\omega_{nb}^{b}=0$, $\left\|\omega_{ib}^{b}-b_{g}\right\|\approx \left\|\omega_{ie}^{b}\right\|\approx 7.3\times 10^{-5}\;\mathrm{rad}/s$ are small amounts. Equation (15) can be simplified as [19]:

(16) $$C_{m}^{b}K\dot{y}=C_{n}^{b}g^{n}+f^{b}-b_{a}.$$

Make derivatives with respect to time on both sides:

(17) $$C_{m}^{b}K\ddot{y}={\dot{f}}^{b}.$$

Take the mode of both sides:

(18) $$k=\pm \left\|\ddot{y}\right\|/\left\|{\dot{f}}^{b}\right\|.$$

In general, the odometer scale factor is positive

(19) $$k=\left\|\ddot{y}\right\|/\left\|{\dot{f}}^{b}\right\|.$$

Rewrite Equation (17) into the following form

(20) $$\frac{{\ddot{y}}_{odo}}{k}\left[\begin{array}{c} -\cos\alpha_{x}\sin\alpha_{z}\\ \cos\alpha_{x}\cos\alpha_{z}\\ \sin\alpha_{x} \end{array}\right]={\dot{f}}^{b}.$$

It is obviously that the odometer scale factor k and IMU misalignment angle $\alpha_{x}$, $\alpha_{z}$ can be calculated from Equations (19) and (20) respectively, and the self-calibration algorithm will be designed in the next section.

## 3. Self-Calibration & Initial Alignment Algorithm

### 3.1. Self-Calibration Algorithm

According to the results of observability analysis in the previous section, it is feasible to construct an ideal observer to estimate the odometer scale factor and IMU misalignment angle based on Equations (19) and (20). Integrating Equation (20) twice over the subinterval $\left[t_{0}\;t\right]$

(21) $$C_{m}^{b}K\alpha \left(t\right)=\beta \left(t\right),$$

where, $\alpha \left(t\right)≜y\left(t\right)-y\left(t_{0}\right)-\dot{y}\left(t_{0}\right)\left(t-t_{0}\right)$, $\beta \left(t\right)=\int_{t_{0}}^{t}f^{b}d\tau -f^{b}\left(t_{0}\right)\left(t-t_{0}\right)$. Compared with Equation (20), this kind of integral form decline the effect of measurement noise. This equation is applied to all the segments that the vehicle has no attitude maneuver and the acceleration is not zero. And the calculation algorithms of the odometer scale factor and IMU misalignment angle are shown as below:

(22) k=‖α(t)‖/‖β(t)‖,

(23) $$\frac{\alpha \left(t\right)}{k}\left[\begin{array}{c} -\cos\alpha_{x}\sin\alpha_{z}\\ \cos\alpha_{x}\cos\alpha_{z}\\ \sin\alpha_{x} \end{array}\right]=\beta \left(t\right).$$

### 3.2. Initial Alignment and Calibration Algorithm

The aim of this section is to figure out the initial alignment and calibration method based on the known odometer scale factor and IMU misalignment angle.

According to the content of last section, $C_{m}^{b}\mathrm{Ky}$ is a known parameter, denoted as:

(24) $$v_{od}^{b}=C_{m}^{b}\mathrm{Ky}.$$

The frozen e-frame at the beginning of the initial alignment is defined as the inertial frame, i.e., i-frame. By the chain rule of the attitude matrix, $C_{n}^{b}\left(t\right)$ at any time satisfies:

(25) $$C_{n}^{b}\left(t\right)=C_{n\left(t\right)}^{b\left(t\right)}=C_{b\left(0\right)}^{b\left(t\right)}C_{n\left(0\right)}^{b\left(0\right)}C_{n\left(t\right)}^{n\left(0\right)}=C_{b\left(0\right)}^{b\left(t\right)}C_{n}^{b}\left(0\right)C_{n\left(t\right)}^{n\left(0\right)},$$

where, $C_{n}^{b}\left(0\right)$ is the initial attitude matrix from n-frame to b-frame, $C_{b\left(0\right)}^{b\left(t\right)}$ and $C_{n\left(t\right)}^{n\left(0\right)}$ encode the attitude changes of the b-frame and n-frame from time 0 to *t* respectively. Their rate equations are:

(26) $${\dot{C}}_{b\left(t\right)}^{b\left(0\right)}=C_{b\left(t\right)}^{b\left(0\right)}\left(\omega_{ib}^{b}-b_{g}\right)\times ,$$

(27) $${\dot{C}}_{n\left(t\right)}^{n\left(0\right)}=C_{n\left(t\right)}^{n\left(0\right)}\left(\omega_{in}^{n}\times \right),$$

where, $\omega_{in}^{n}$ denotes the angular velocity of n-frame with respect to the inertial frame, i.e., $\omega_{in}^{n}=\omega_{ie}^{n}+\omega_{en}^{n}$.

Substituting Equations (24) and (25) into Equation (15), and rewriting as:

(28) $$C_{b\left(t\right)}^{b\left(0\right)}\left({\dot{v}}_{od}^{b}+\left(\omega_{ib}^{b}-b_{g}+\omega_{ie}^{b}\right)\times v_{od}^{b}-\left[{\dot{\omega }}_{nb}^{b}+\left(\omega_{ib}^{b}-b_{g}+\omega_{ie}^{b}\right)\times \omega_{nb}^{b}\right]\times l^{b}-\left(f^{b}-b_{a}\right)\right)=C_{n}^{b}\left(0\right)C_{n\left(t\right)}^{n\left(0\right)}g^{n}.$$

Make integration with respect to time on both sides of Equation (28):

(29) $$\begin{array}{l} \int_{0}^{t}C_{b\left(t\right)}^{b\left(0\right)}\left[{\dot{v}}_{od}^{b}+\left(\omega_{ib}^{b}-b_{g}+\omega_{ie}^{b}\right)\times v_{od}^{b}\right]dt\\ -\int_{0}^{t}C_{b\left(t\right)}^{b\left(0\right)}\left[{\dot{\omega }}_{nb}^{b}+\left(\omega_{ib}^{b}-b_{g}+\omega_{ie}^{b}\right)\times \omega_{nb}^{b}\right]\times dtl^{b}\\ -\int_{0}^{t}C_{b\left(t\right)}^{b\left(0\right)}\left(f^{b}-b_{a}\right)dt\\ =C_{n}^{b}\left(0\right)\int_{0}^{t}C_{n\left(t\right)}^{n\left(0\right)}g^{n}dt \end{array},$$

where:

(30) $$\int_{0}^{t}C_{b\left(t\right)}^{b\left(0\right)}{\dot{v}}_{od}^{b}dt=C_{b\left(t\right)}^{b\left(0\right)}v_{od}^{b}-v_{od}^{b}\left(0\right)-\int_{0}^{t}C_{b\left(t\right)}^{b\left(0\right)}\left(\omega_{ib}^{b}-b_{g}\right)\times v_{od}^{b}dt,$$

(31) $$\int_{0}^{t}C_{b\left(t\right)}^{b\left(0\right)}\left({\dot{\omega }}_{nb}^{b}\times \right)dt=C_{b\left(t\right)}^{b\left(0\right)}\left(\omega_{nb}^{b}\times \right)-\left(\omega_{nb}^{b}\left(0\right)\times \right)-\int_{0}^{t}C_{b\left(t\right)}^{b\left(0\right)}\left(\omega_{ib}^{b}-b_{g}\right)\times \omega_{nb}^{b}\times dt.$$

Substituting the above equations into Equation (29)

(32) $$\begin{array}{l} C_{b\left(t\right)}^{b\left(0\right)}v_{od}^{b}-v_{od}^{b}\left(0\right)+\int_{0}^{t}C_{b\left(t\right)}^{b\left(0\right)}\omega_{ie}^{b}\times v_{od}^{b}dt\\ -\left(C_{b\left(t\right)}^{b\left(0\right)}\left(\omega_{nb}^{b}\times \right)-\left(\omega_{nb}^{b}\left(0\right)\times \right)+\int_{0}^{t}C_{b\left(t\right)}^{b\left(0\right)}\omega_{ie}^{b}\times \omega_{nb}^{b}\times dt\right)l^{b}\\ -\int_{0}^{t}C_{b\left(t\right)}^{b\left(0\right)}\left(f^{b}-b_{a}\right)dt\\ =C_{n}^{b}\left(0\right)\int_{0}^{t}C_{n\left(t\right)}^{n\left(0\right)}g^{n}dt \end{array}.$$

During the attitude maneuvering, $\omega_{ie}^{b}$ is much smaller amount than $\omega_{nb}^{b}$. Equation (32) can be simplified as:

(33) $$C_{b\left(t\right)}^{b\left(0\right)}v_{od}^{b}-v_{od}^{b}\left(0\right)-\left(C_{b\left(t\right)}^{b\left(0\right)}\omega_{nb}^{b}-\omega_{nb}^{b}\left(0\right)\right)\times l^{b}-\int_{0}^{t}C_{b\left(t\right)}^{b\left(0\right)}\left(f^{b}-b_{a}\right)dt=C_{n}^{b}\left(0\right)\int_{0}^{t}C_{n\left(t\right)}^{n\left(0\right)}g^{n}dt,$$

where, $C_{b\left(t\right)}^{b\left(0\right)}$ can be simplified in the first order as:

(34) $$\begin{array}{ll} C_{b\left(t\right)}^{b\left(0\right)} & =C_{b\left(T\right)}^{b\left(0\right)}\cdots C_{b\left(MT\right)}^{b\left(\left(M-1\right)T\right)}\approx \prod_{i=1}^{M}\left(I+\Theta_{i}-Tb_{g}\times \right)\\ & \approx {\tilde{C}}_{b\left(t_{M}\right)}^{b\left(0\right)}-T\sum_{i=1}^{M}\left[\prod_{j=1}^{i-1}\left(I+\Theta_{j}\right)\right]\times \left(b_{g}\times \right)\left[\prod_{j=i+1}^{M}\left(I+\Theta_{j}\right)\right]\\ & \approx {\tilde{C}}_{b\left(t_{M}\right)}^{b\left(0\right)}-MTb_{g}\times \end{array},$$

where, ${\tilde{C}}_{b\left(t_{M}\right)}^{b\left(0\right)}$ denotes the error-contaminated body matrix computed by $\omega_{ib}^{b}$ and $\Theta_{i}$ is the skew symmetric matrix formed by the error-contaminated incremental rotation vector during the update interval [*t_i−_*_1_ *t_i_*]. For notational brevity, the $C_{b\left(t_{M}\right)}^{b\left(0\right)}$ is used instead of ${\tilde{C}}_{b\left(t_{M}\right)}^{b\left(0\right)}$ in the later sections. *M* is the current sequence, and T is the integral period.

Substituting Equation (34) into the left integral of Equation (33):

(35) $$\begin{array}{ll} \int_{0}^{t}C_{b\left(t\right)}^{b\left(0\right)}\left(f^{b}-b_{a}\right)dt & =\sum_{k=0}^{M-1}\int_{t_{k}}^{t_{k+1}}C_{b\left(t\right)}^{b\left(0\right)}\left(f^{b}-b_{a}\right)dt\\ & =\sum_{k=0}^{M-1}C_{b\left(t_{k}\right)}^{b\left(0\right)}\int_{t_{k}}^{t_{k+1}}C_{b\left(t\right)}^{b\left(t_{k}\right)}\left(f^{b}-b_{a}\right)dt\\ & =\sum_{k=0}^{M-1}\left(C_{b\left(t_{k}\right)}^{b\left(0\right)}-kTb_{g}\times \right)\int_{t_{k}}^{t_{k+1}}\left(I+\left(\int_{t_{k}}^{t}\left(\omega_{ib}^{b}-b_{g}\right)d\tau \right)\times \right)\left(f^{b}-b_{a}\right)dt \end{array},$$

where, the incremental integral above can be approximated using the two-sample correction by:

(36) $$\begin{array}{l} \int_{t_{k}}^{t_{k+1}}\left(I+\left(\int_{t_{k}}^{t}\left(\omega_{ib}^{b}-b_{g}\right)d\tau \right)\times \right)\left(f^{b}-b_{a}\right)dt\\ =\int_{t_{k}}^{t_{k+1}}\left(I+\left(\int_{t_{k}}^{t}\omega_{ib}^{b}d\tau \right)\times \right)f^{b}dt-\int_{t_{k}}^{t_{k+1}}\left(I+\left(\int_{t_{k}}^{t}\omega_{ib}^{b}d\tau \right)\times \right)dtb_{a}\\ \;-b_{g}\times \int_{t_{k}}^{t_{k+1}}\left(t-t_{k}\right)\left(f^{b}-b_{a}\right)dt\\ =\Delta v_{1}+\Delta v_{2}+\frac{1}{2}\left(\Delta \theta_{1}+\Delta \theta_{2}\right)\times \left(\Delta v_{1}+\Delta v_{2}\right)+\frac{2}{3}\left(\Delta \theta_{1}\times \Delta v_{2}+\Delta v_{1}\times \Delta \theta_{2}\right)\\ \;-\left[TI+\frac{T}{6}\left(5\Delta \theta_{1}+\Delta \theta_{2}\right)\times \right]b_{a}+\left[\frac{T}{6}\left(\Delta v_{1}+5\Delta v_{2}\right)-\frac{T^{2}}{2}b_{a}\right]\times b_{g} \end{array},$$

where, $\Delta v_{1}$ and $\Delta v_{2}$ are the first and the second samples of the incremental velocity measured by accelerometers, $\Delta \theta_{1}$ and $\Delta \theta_{2}$ are the first and the second samples of the incremental angle measured by gyroscopes during the update interval $\left[t_{k}\;t_{k+1}\right]$, respectively. Substituting Equation (36) into (35) and rewriting the equation by neglecting those products of IMU biases higher than the first order, we have:

(37) $$\begin{array}{l} \int_{0}^{t}C_{b\left(t\right)}^{b\left(0\right)}\left(f^{b}-b_{a}\right)dt\\ \approx \sum_{k=0}^{M-1}C_{b\left(t_{k}\right)}^{b\left(0\right)}\left[\Delta v_{1}+\Delta v_{2}+\frac{1}{2}\left(\Delta \theta_{1}+\Delta \theta_{2}\right)\times \left(\Delta v_{1}+\Delta v_{2}\right)+\frac{2}{3}\left(\Delta \theta_{1}\times \Delta v_{2}+\Delta v_{1}\times \Delta \theta_{2}\right)\right]\\ -\sum_{k=0}^{M-1}C_{b\left(t_{k}\right)}^{b\left(0\right)}\left[TI+\frac{T}{6}\left(5\Delta \theta_{1}+\Delta \theta_{2}\right)\times \right]b_{a}+\sum_{k=0}^{M-1}C_{b\left(t_{k}\right)}^{b\left(0\right)}\left[\frac{T}{6}\left(\Delta v_{1}+5\Delta v_{2}\right)\right]\times b_{g}\\ +\sum_{k=0}^{M-1}\left[\Delta v_{1}+\Delta v_{2}+\frac{1}{2}\left(\Delta \theta_{1}+\Delta \theta_{2}\right)\times \left(\Delta v_{1}+\Delta v_{2}\right)+\frac{2}{3}\left(\Delta \theta_{1}\times \Delta v_{2}+\Delta v_{1}\times \Delta \theta_{2}\right)\right]\times kTb_{g} \end{array}.$$

Discretize the integral on the right of the Equation (28) as:

(38) $$\int_{0}^{t}C_{n\left(t\right)}^{n\left(0\right)}g^{n}dt=\sum_{k=0}^{M-1}\int_{t_{k}}^{t_{k+1}}C_{n\left(t\right)}^{n\left(0\right)}g^{n}dt=\sum_{k=0}^{M-1}C_{n\left(t_{k}\right)}^{n\left(0\right)}\int_{t_{k}}^{t_{k+1}}C_{n\left(t\right)}^{n\left(t_{k}\right)}g^{n}dt.$$

The n-frame rate $\omega_{in}^{n}$ changes much slower than the body rate $\omega_{ib}^{b}$, so $C_{n\left(t\right)}^{n\left(t_{k}\right)}$ can be approximated as

(39) $$\begin{array}{ll} C_{n\left(t\right)}^{n\left(t_{k}\right)} & =I+\frac{\sin\left(\left\|\varphi_{n}\right\|\right)}{\left\|\varphi_{n}\right\|}\left(\varphi_{n}\times \right)+\frac{1-\cos\left(\left\|\varphi_{n}\right\|\right)}{{\left\|\varphi_{n}\right\|}^{2}}{\left(\varphi_{n}\times \right)}^{2}\\ & \approx I+\left(\varphi_{n}\times \right) \end{array},$$

where, $\varphi_{n}\approx \int_{t_{k}}^{t}\omega_{in}^{n}dt\approx \left(t-t_{k}\right)\omega_{in}^{n}$ denotes the rotation vector of n-frame from *t_k_* to the current time *t*. The integral can be approximated by:

(40) $$\begin{array}{l} \int_{0}^{t}C_{n\left(t\right)}^{n\left(0\right)}g^{n}dt\approx \sum_{k=0}^{M-1}C_{n\left(t_{k}\right)}^{n\left(0\right)}\int_{t_{k}}^{t_{k+1}}\left(I+\left(t-t_{k}\right)\omega_{in}^{n}\times \right)g^{n}dt\\ \;=\sum_{k=0}^{M-1}C_{n\left(t_{k}\right)}^{n\left(0\right)}\left(TI+\frac{T^{2}}{2}\omega_{in}^{n}\times \right)g^{n} \end{array}.$$

Substituting Equations (36), (37) and (40) into the left integral of Equation (33):

(41) $$\alpha_{M}+\gamma_{M}l^{b}+\chi_{M}b_{a}+\lambda_{M}b_{g}=C_{n}^{b}\left(0\right)\beta_{M},$$

where the symbols in Equation (41) are defined as follows:

$$\Delta v=\Delta v_{1}+\Delta v_{2}+\frac{1}{2}\left(\Delta \theta_{1}+\Delta \theta_{2}\right)\times \left(\Delta v_{1}+\Delta v_{2}\right)+\frac{2}{3}\left(\Delta \theta_{1}\times \Delta v_{2}+\Delta v_{1}\times \Delta \theta_{2}\right),$$

$$\alpha_{M}=C_{b\left(t_{M}\right)}^{b\left(0\right)}v_{od}^{b}-v_{od}^{b}\left(0\right)-\sum_{k=0}^{M-1}C_{b\left(t_{k}\right)}^{b\left(0\right)}\Delta v,$$

$$\chi_{M}=\sum_{k=0}^{M-1}C_{b\left(t_{k}\right)}^{b\left(0\right)}\left[TI+\frac{T}{6}\left(5\Delta \theta_{1}+\Delta \theta_{2}\right)\times \right],$$

$$\lambda_{M}=MT\left(v_{od}^{b}\times \right)-\sum_{k=0}^{M-1}\left(C_{b\left(t_{k}\right)}^{b\left(0\right)}\left[\frac{T}{6}\left(\Delta v_{1}+5\Delta v_{2}\right)\times \right]+kT\Delta v\times \right),$$

$$\gamma_{M}=-\left(C_{b\left(t\right)}^{b\left(0\right)}\omega_{ib}^{b}\times -\omega_{ib}^{b}\left(0\right)\times \right),$$

$$\beta_{M}=\sum_{k=0}^{M-1}C_{n\left(t_{k}\right)}^{n\left(0\right)}\left(TI+\frac{T^{2}}{2}\omega_{in}^{n}\times \right)g^{n}.$$

### 3.3. Optimization-Based Attitude and Parameter Estimation

In this section, Equation (41) will be posed as a constrained minimization problem to estimate the attitude and other parameters. The attitude matrix $C_{b}^{n}\left(0\right)$ is replaced by the four-element unit quaternion $q={\left[s\;\eta \right]}^{T}$, where s is the scalar part and η is the vector part. The relationship between the unit quaternion and the attitude matrix is [1]:

(42) $$C_{b}^{n}\left(0\right)=\left(s^{2}-\eta^{T}\eta \right)I+2\eta \eta^{T}+2s\left(\eta \times \right).$$

Define the quaternion multiplication as:

(43) $$q_{1}\circ q_{2}=\overset{+}{\left[q_{1}\right]}\left[\begin{array}{l} s_{2}\\ \eta_{2} \end{array}\right]=\overset{-}{\left[q_{2}\right]}\left[\begin{array}{l} s_{1}\\ \eta_{1} \end{array}\right],$$

where, $\overset{+}{\left[q\right]}=\left[\begin{array}{cc} s & -\eta^{T}\\ \eta & sI+\left(\eta \times \right) \end{array}\right]$, $\overset{-}{\left[q\right]}=\left[\begin{array}{cc} s & -\eta^{T}\\ \eta & sI-\left(\eta \times \right) \end{array}\right]$.

Then Equation (41) is equivalent to:

(44) $$\alpha_{M}+\gamma_{M}l^{b}+\chi_{M}b_{a}+\lambda_{M}b_{g}=q^{*}\circ \beta_{M}\circ q.$$

Multiply both sides by q

(45) $$q\circ \left(\alpha_{M}+\gamma_{M}l^{b}+\chi_{M}b_{a}+\lambda_{M}b_{g}\right)=\beta_{M}\circ q.$$

According to Equation (43), Equation (45) can be rewritten as:

(46) $$0=\left(\overset{-}{\left[\alpha_{M}\right]}-\overset{+}{\left[\beta_{M}\right]}\right)q+\overset{+}{\left[q\right]}\left(\gamma_{M}l^{b}+\chi_{M}b_{a}+\lambda_{M}b_{g}\right)≜\pi .$$

Since the magnitude of the unit quaternion is 1, we can pose the problem as a unit quaternion-constrained optimization [21,29]:

(47) $$\underset{q,l^{b},b_{a},b_{g}}{\min}\sum_{M}\pi^{T}\pi ,s.t.q^{T}q=1.$$

Ignoring the IMU biases and the lever arm, it is reduced to the Wahba problem [30], which is famous in attitude determination:

(48) $$\underset{q}{\min}\sum_{M}q^{T}{\left(\overset{-}{\left[\alpha_{M}\right]}-\overset{+}{\left[\beta_{M}\right]}\right)}^{T}\left(\overset{-}{\left[\alpha_{M}\right]}-\overset{+}{\left[\beta_{M}\right]}\right)q,s.t.q^{T}q=1.$$

Denote $K=\sum_{M}{\left(\overset{-}{\left[\alpha_{M}\right]}-\overset{+}{\left[\beta_{M}\right]}\right)}^{T}\left(\overset{-}{\left[\alpha_{M}\right]}-\overset{+}{\left[\beta_{M}\right]}\right)$, the solution of Wahba problem is the eigenvectors belonging to the minimum eigenvalues of the matrix K. And the solution will be taken as the initial angle of the following Newton-Lagrange method.

The iterative Newton-Lagrange method is chosen to tackle the nonlinearly constrained optimization problem, and the Lagrangian equation for the problem (47) is defined as [31]:

(49) $$L\left(x,\mu \right)=\sum_{M}\pi^{T}\pi +\mu \left(q^{T}q-1\right),$$

where, $x≜{\left[\begin{array}{cccc} q^{T} & l^{b}{}^{T} & b_{a}{}^{T} & b_{g}{}^{T} \end{array}\right]}^{T}$, μ is the Lagrange multiplier. The iterative algorithm is given as below:

(50) $$\left[\begin{array}{l} x_{k+1}\\ \mu_{k+1} \end{array}\right]=\left[\begin{array}{l} x_{k}\\ \mu_{k} \end{array}\right]+\left[\begin{array}{l} \Delta x\\ \Delta \mu \end{array}\right],$$

where, Δx and Δμ are calculated by:

(51) $$\nabla^{2}L\left(x,\mu \right)\left[\begin{array}{l} \Delta x\\ \Delta \mu \end{array}\right]=-\nabla L\left(x_{k},\mu_{k}\right).$$

Namely:

(52) $$\left[\begin{array}{cc} \nabla_{xx}^{2}L\left(x_{k},\mu_{k}\right) & \nabla_{x\mu }^{2}L\left(x_{k},\mu_{k}\right)\\ \nabla_{x\mu }^{2}L{\left(x_{k},\mu_{k}\right)}^{T} & 0 \end{array}\right]\left[\begin{array}{l} \Delta x\\ \Delta \mu \end{array}\right]=-\left[\begin{array}{c} \nabla_{x}L\left(x_{k},\mu_{k}\right)\\ q^{T}q-1 \end{array}\right].$$

The first and the second derivatives of $L\left(x_{k},\mu_{k}\right)$ are expressed as follows:

$$\nabla_{x}L\left(x_{k},\mu_{k}\right)=\sum_{M}J-2\mu {\left[\begin{array}{cccc} q^{T} & 0_{1\times 3} & 0_{1\times 3} & 0_{1\times 3} \end{array}\right]}^{T},$$

$$\nabla_{x\mu }^{2}L\left(x_{k},\mu_{k}\right)=-2{\left[\begin{array}{cccc} q^{T} & 0_{1\times 3} & 0_{1\times 3} & 0_{1\times 3} \end{array}\right]}^{T},$$

$$\nabla_{xx}^{2}L\left(x_{k},\mu_{k}\right)=\sum_{M}H-2\mu diag\left\{{\left[\begin{array}{cc} 1_{1\times 4} & 0_{1\times 9} \end{array}\right]}^{T}\right\},$$

where, $J≜{\left[\begin{array}{cccc} J_{1}^{T} & J_{2}^{T} & J_{3}^{T} & J_{4}^{T} \end{array}\right]}^{T}$ is the Jacobian matrix of $\pi^{T}\pi$ with:

$$\begin{array}{l} J_{1}=2{\left(\overset{-}{\left[\alpha_{M}\right]}-\overset{+}{\left[\beta_{M}\right]}\right)}^{T}\left(\overset{-}{\left[\alpha_{M}\right]}-\overset{+}{\left[\beta_{M}\right]}\right)q\\ +2\left(\overset{-}{{\left[\gamma_{M}l^{b}+\chi_{M}b_{a}+\lambda_{M}b_{g}\right]}^{T}}\left(\overset{-}{\left[\alpha_{M}\right]}-\overset{+}{\left[\beta_{M}\right]}\right)+{\left(\overset{-}{\left[\alpha_{M}\right]}-\overset{+}{\left[\beta_{M}\right]}\right)}^{T}\overset{-}{\left[\gamma_{M}l^{b}+\chi_{M}b_{a}+\lambda_{M}b_{g}\right]}\right)q \end{array},$$

$$J_{2}=2\gamma_{M}^{T}\overset{+}{{\left[q\right]}^{T}}\left(\overset{-}{\left[\alpha_{M}\right]}-\overset{+}{\left[\beta_{M}\right]}\right)q+2\gamma_{M}^{T}\left(\gamma_{M}l^{b}+\chi_{M}b_{a}+\lambda_{M}b_{g}\right),$$

$$J_{3}=2\chi_{M}^{T}\overset{+}{{\left[q\right]}^{T}}\left(\overset{-}{\left[\alpha_{M}\right]}-\overset{+}{\left[\beta_{M}\right]}\right)q+2\chi_{M}^{T}\left(\gamma_{M}l^{b}+\chi_{M}b_{a}+\lambda_{M}b_{g}\right),$$

$$J_{4}=2\lambda_{M}^{T}\overset{+}{{\left[q\right]}^{T}}\left(\overset{-}{\left[\alpha_{M}\right]}-\overset{+}{\left[\beta_{M}\right]}\right)q+2\lambda_{M}^{T}\left(\gamma_{M}l^{b}+\chi_{M}b_{a}+\lambda_{M}b_{g}\right),$$

and $H≜\left[\begin{array}{cccc} H_{11} & H_{12} & H_{13} & H_{14}\\ H_{12}^{T} & H_{22} & H_{23} & H_{24}\\ H_{13}^{T} & H_{23}^{T} & H_{33} & H_{34}\\ H_{14}^{T} & H_{24}^{T} & H_{34}^{T} & H_{44} \end{array}\right]$ is the Hessian matrix of $\pi^{T}\pi$ with:

$$\begin{array}{ll} H_{11} & =2{\left(\overset{-}{\left[\alpha_{M}\right]}-\overset{+}{\left[\beta_{M}\right]}\right)}^{T}\left(\overset{-}{\left[\alpha_{M}\right]}-\overset{+}{\left[\beta_{M}\right]}\right)\\ & +2\left(\overset{-}{{\left[\gamma_{M}l^{b}+\chi_{M}b_{a}+\lambda_{M}b_{g}\right]}^{T}}\left(\overset{-}{\left[\alpha_{M}\right]}-\overset{+}{\left[\beta_{M}\right]}\right)+{\left(\overset{-}{\left[\alpha_{M}\right]}-\overset{+}{\left[\beta_{M}\right]}\right)}^{T}\overset{-}{\left[\gamma_{M}l^{b}+\chi_{M}b_{a}+\lambda_{M}b_{g}\right]}\right) \end{array},$$

$$H_{12}=2\left({\left(\overset{-}{\left[\alpha_{M}\right]}-\overset{+}{\left[\beta_{M}\right]}\right)}^{T}\overset{+}{\left[q\right]}-\overset{+}{\left[\left(\overset{-}{\left[\alpha_{M}\right]}-\overset{+}{\left[\beta_{M}\right]}\right)q\right]}\right)\gamma_{M},$$

$$H_{13}=2\left({\left(\overset{-}{\left[\alpha_{M}\right]}-\overset{+}{\left[\beta_{M}\right]}\right)}^{T}\overset{+}{\left[q\right]}-\overset{+}{\left[\left(\overset{-}{\left[\alpha_{M}\right]}-\overset{+}{\left[\beta_{M}\right]}\right)q\right]}\right)\chi_{M},$$

$$H_{14}=2\left({\left(\overset{-}{\left[\alpha_{M}\right]}-\overset{+}{\left[\beta_{M}\right]}\right)}^{T}\overset{+}{\left[q\right]}-\overset{+}{\left[\left(\overset{-}{\left[\alpha_{M}\right]}-\overset{+}{\left[\beta_{M}\right]}\right)q\right]}\right)\lambda_{M},$$

$$H_{22}=2\gamma_{M}^{T}\gamma_{M},\;H_{23}=2\gamma_{M}^{T}\chi_{M},\;H_{24}=2\gamma_{M}^{T}\lambda_{M},$$

$$H_{33}=2\chi_{M}^{T}\chi_{M},\;H_{34}=2\chi_{M}^{T}\lambda_{M},\;H_{44}=2\lambda_{M}^{T}\lambda_{M}.$$

## 4. Simulation and Experiment

To verify the performance of the proposed optimization-based initial alignment algorithm, simulations and experiments are performed in this section.

### 4.1. Simulation and Analysis

The vehicle is assumed to be located at medium latitude 45° and equipped with a triad of gyroscopes (drift 0.02°/h, noise 0.002°/h/$\sqrt{\mathrm{Hz}}$) and accelerometers (bias 100 μg, noise 10 μg/$\sqrt{\mathrm{Hz}}$) at a sampling rate 100 Hz. The IMU misalignment angle is [20′ 10′ 30′]^T^. The odometer is displaced from the IMU by the lever arm $l^{b}={\left[1\;3.2\;-0.5\right]}^{T}$ in meters and the odometer scale factor error is 0.002. White noise of velocity (standard variance 0.02 m/s) is simulated in odometer measurements. The initial attitude error is [1° 1° 10°]^T^, the initial position error is 10m for each direction in latitude, longitude and height.

**Figure 2:**
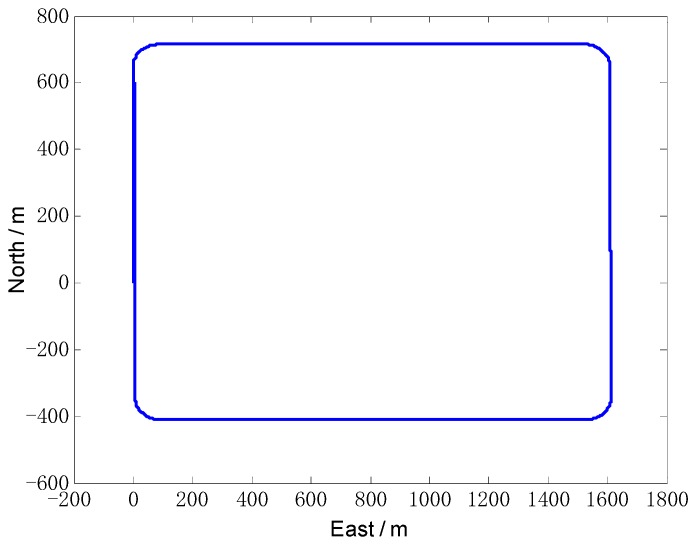
The vehicle’s simulation trajectory.

**Figure 3:**
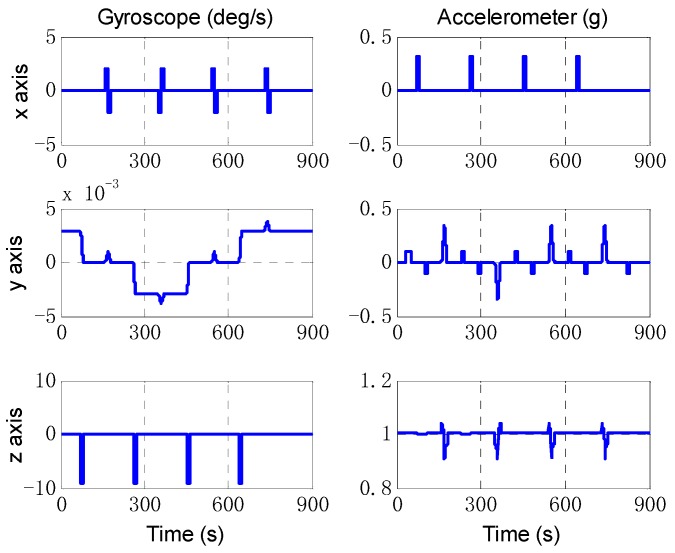
The IMU data of typical motions simulation. The left three figures are the angular rate measured by gyroscopes, the right three figures are the specific forces.

Firstly, the simulations are designed to mimic the typical motions of a land vehicle, the vehicle trajectory is designed as follows. The total simulation time is 1000 s. The maneuver mode includes the accelerating, turning, pitching and slowing down. The vehicle’s running trajectory is shown in Figure 2, the outputs of IMU are shown in Figure 3.

**Figure 4:**
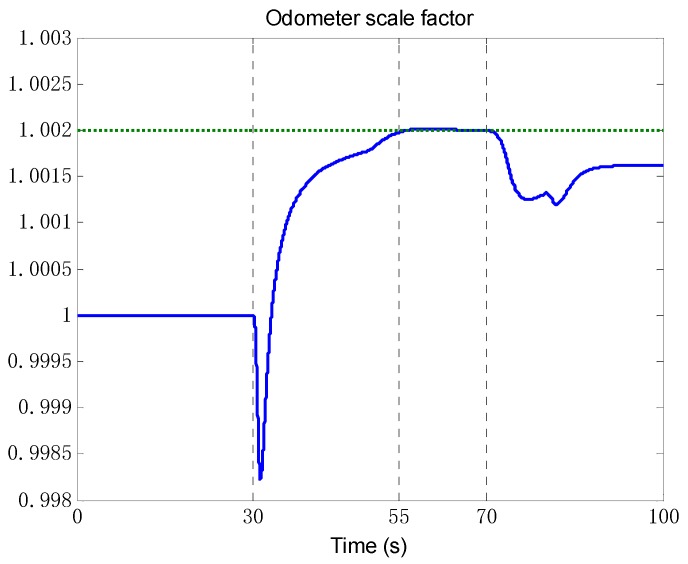
The estimation of odometer scale factor. The blue solid line denotes the estimation by self-calibration algorithm, and the green dashed line denotes the truth value.

**Figure 5:**
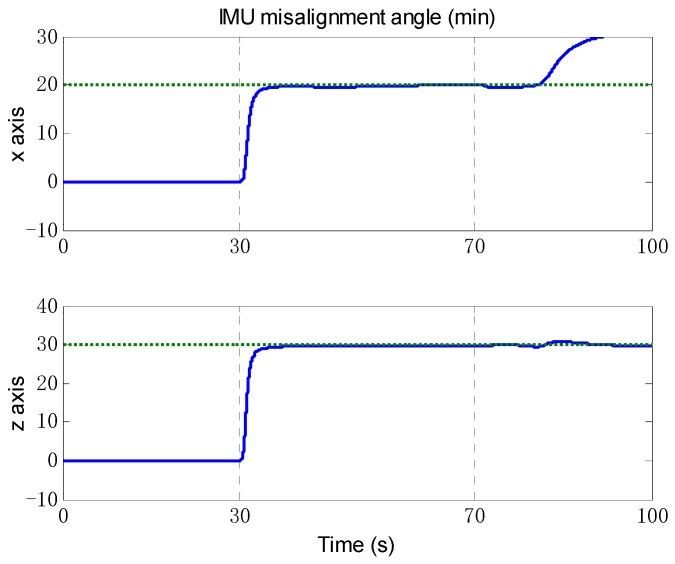
The estimation of IMU misalignment angle. The blue solid line denotes the estimation by self-calibration algorithm, and the green dashed line denotes the truth value. The upper figure is the misalignment angle of *x* axis, and the bottom figure is the misalignment angle of *z* axis.

According to the results of observability analysis in the previous section, the self-calibration algorithm is applied to all the segments that the vehicle has no attitude maneuver and the acceleration is not zero. The trajectory data of the first 100 s are used for the simulation. As shown in Figure 4, the odometer scale factor is effectively estimated at about 55 s after the vehicle accelerating. And the IMU misalignment angle in Figure 5 is also effectively estimated once the vehicle starts to move at 30 s. As expected, the estimated result deviates from the truth value after the vehicle turning at 70 s, because the applicable conditions of the algorithm are not satisfied. The estimated values *k* = 1.00198,$\alpha_{x}=20.02^{\prime }$, $\alpha_{z}=29.85^{\prime }$ will be considered to be known states in the following simulations. 

**Figure 6:**
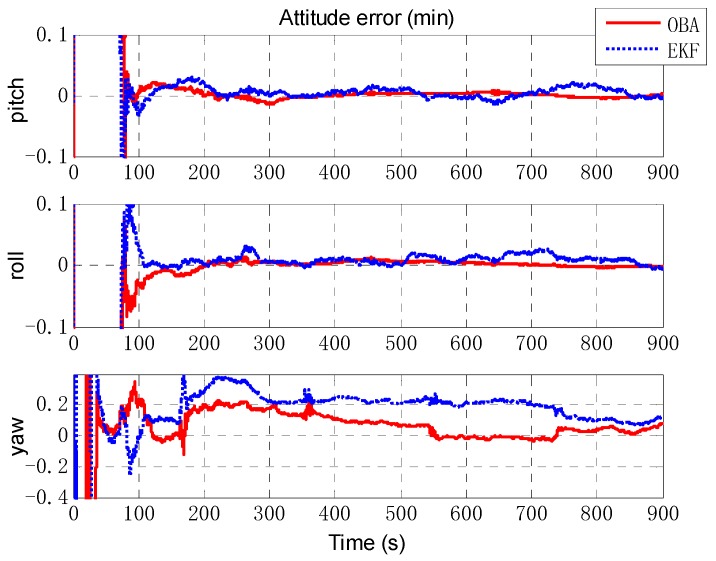
The estimation of attitude error. The red solid line denotes the estimation by OBA, and the blue dashed line denotes the estimation by EKF. The three figures are the attitude errors of pitch, roll and yaw respectively.

**Figure 7:**
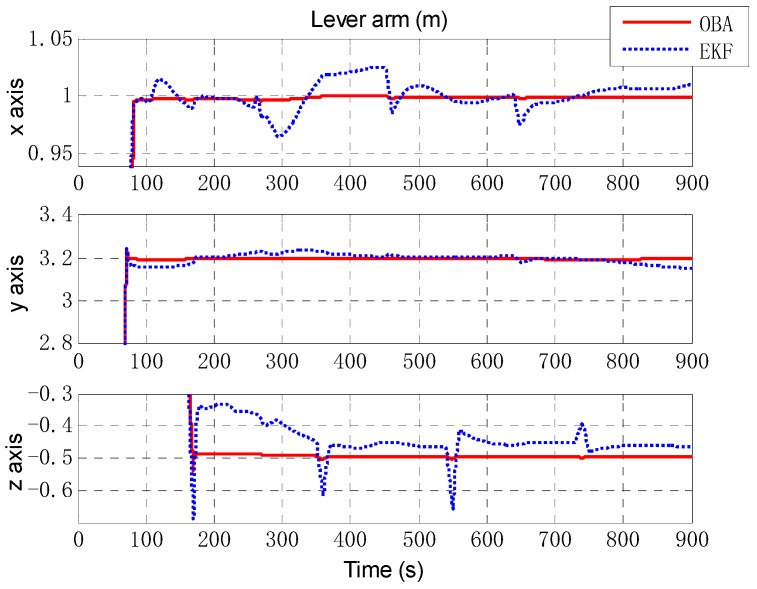
The estimation of lever arm. The red solid line denotes the estimation by OBA, and the blue dashed line denotes the estimation by EKF. The three figures are the lever arm of *x* axis, *y* axis and *z* axis respectively.

Next, an extended Kalman filter (EKF) is implemented as a comparison of the proposed optimization-based alignment (OBA) method. Figure 6 presents the alignment result of attitude error by EKF (the blue dashed line) and OBA (the rad solid line). Roll and pitch gradually converge after 70 s (turning), and the convergence accuracy is better than 0.01′. And due to the large initial error setting, the convergence of the yaw is relatively slow and accuracy is about 0.1′. For the estimation of attitude error, the proposed OBA method is relatively better than EKF as shown in Table 1. The estimation of the lever arm is shown in Figure 7. For horizontal arm (*x* axis and *y* axis), the EKF method is expected to converge rapidly after the course turn, and the vertical arm (*z* axis) converges gradually after pitching. Compared with EKF, the convergence of OBA method is more quickly and the estimation accuracy is much higher. To present the estimate results clearly, the estimate errors are listed in Table 1.

**Figure 8:**
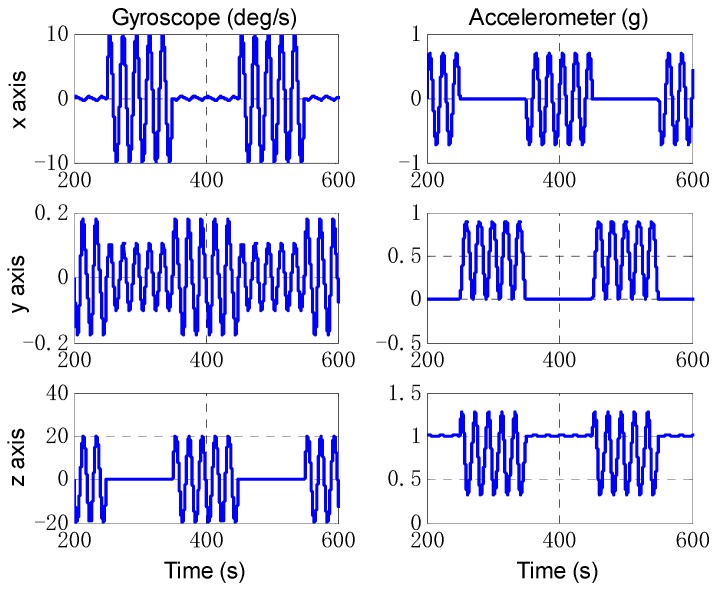
The IMU outputs of angular motion simulation. The left three figures are the angular rate measured by gyroscopes, the right three figures are the specific forces measured by accelerometers.

**Figure 9:**
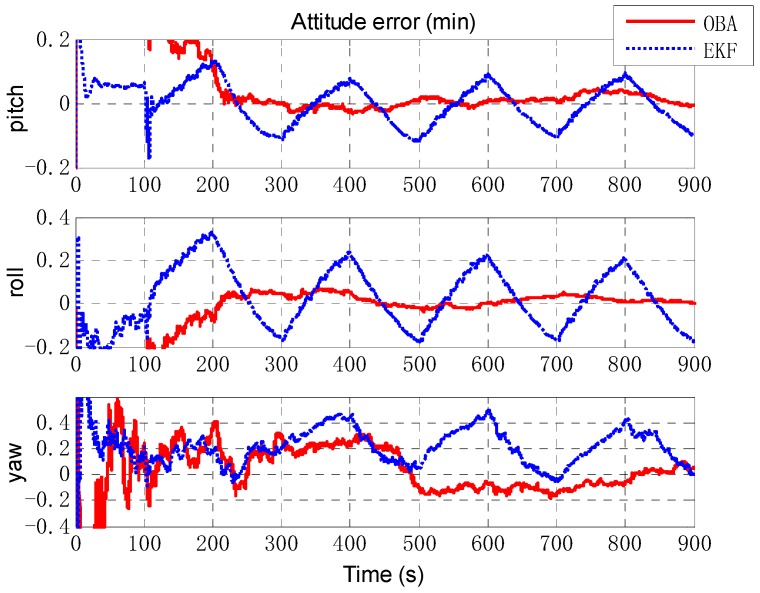
The estimation of attitude error. The red solid line denotes the estimation by OBA, and the blue dashed line denotes the estimation by EKF. The three figures are the attitude errors of pitch, roll and yaw respectively.

**Figure 10:**
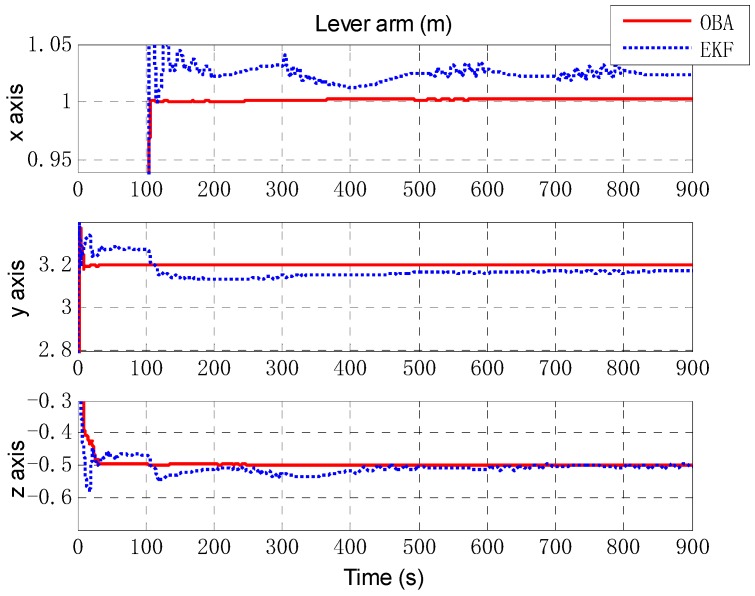
The estimation of lever arm. The red solid line denotes the estimation by OBA, and the blue dashed line denotes the estimation by EKF. The three figures are the lever arm of *x* axis, *y* axis and *z* axis respectively.

Then, we designed a trajectory with alternating yawing and pitching motion, and the IMU outputs are shown in Figure 8. The estimate results of attitude are shown in Figure 9. It can be seen that the estimation of OBA is similar to the last simulation, but the estimate results of EKF vary obviously with the oscillation amplitude about 0.4′. The estimation of lever arm is shown in Figure 10, and the estimate result of EKF has obvious oscillations with the attitude motion too. The estimate errors of EKF and OBA are listed in Table 2. It is clearly that the EKF is susceptible to disturbance of angular motions, while OBA is hardly affected. The OBA algorithm can track attitude motion and it is inherently not influenced by any angular motions.

### 4.2. Experiment and Analysis

**Figure 11:**
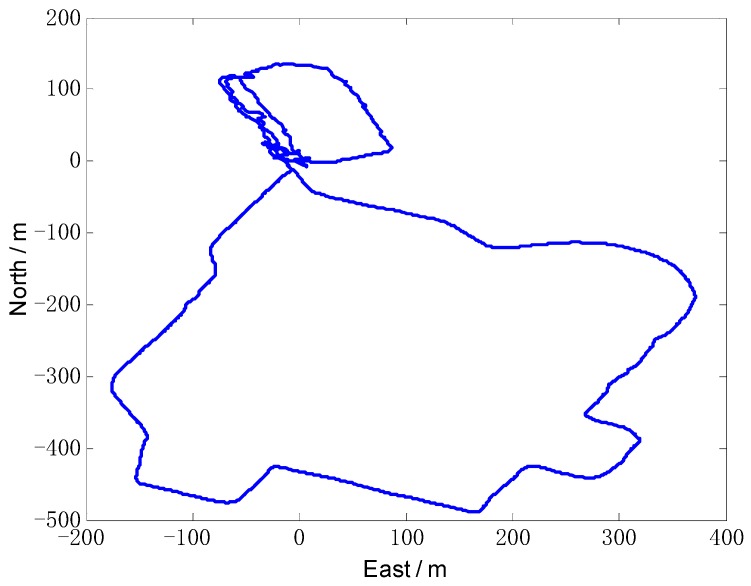
The vehicle test trajectory measured by GPS.

**Figure 12:**
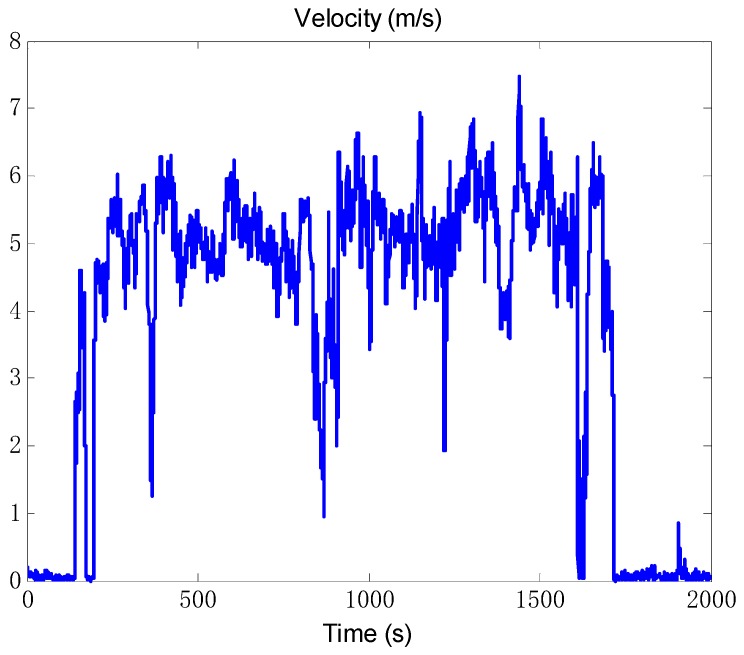
The vehicle test velocity measured by odometer.

A vehicle test was conducted to validate the actual performance of the proposed algorithm. The SINS/odometer system parameters are the same with the simulation condition. A high-precision GPS equipment was chosen as position reference, with the position accuracy less than 3 m and the velocity accuracy 0.1 m/s. And the attitude reference was given by SINS/GPS integrated navigation system. The vehicle test trajectory is shown in Figure 11, and the velocity measured by odometer is shown in Figure 12. 

**Figure 13:**
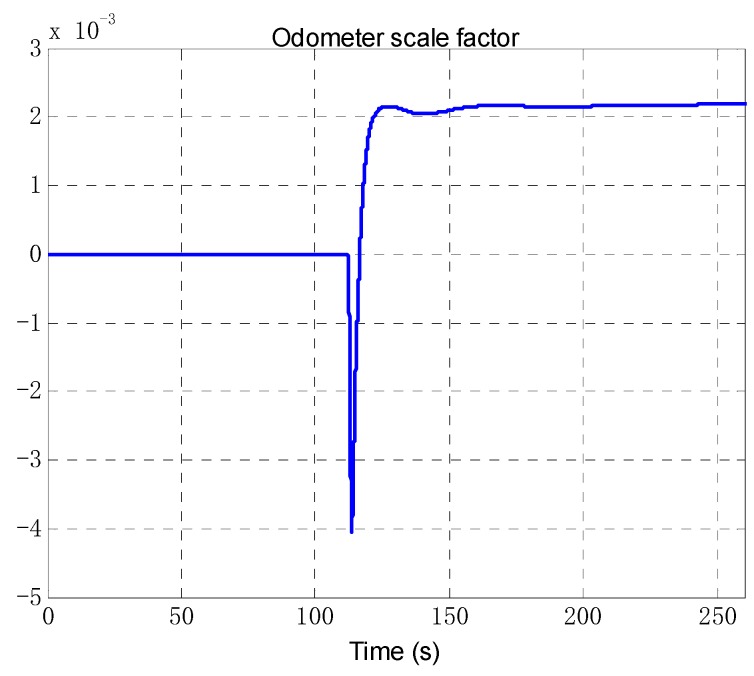
The estimation of odometer scale factor.

**Figure 14:**
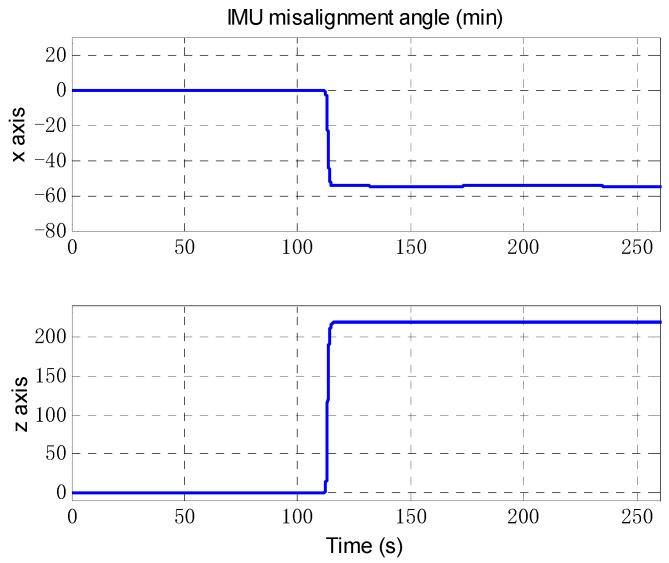
The estimation of IMU misalignment angle. The upper figure is the misalignment angle of *x* axis, and the bottom figure is the misalignment angle of *z* axis.

**Figure 15:**
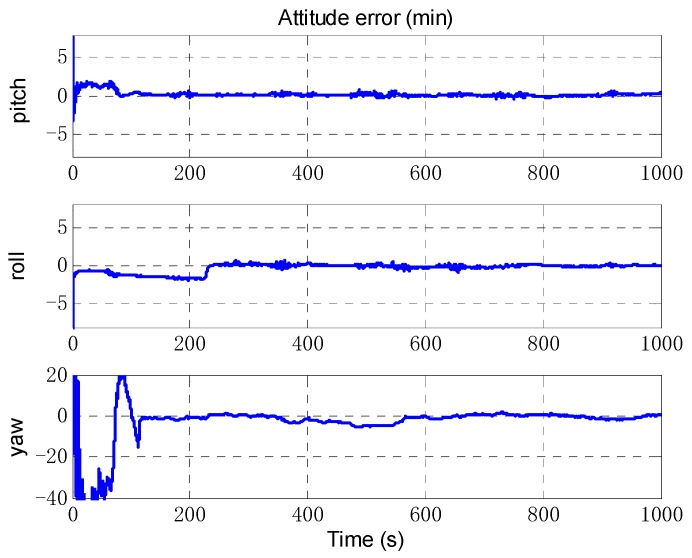
The estimation of attitude error.

The odometer scale factor and IMU misalignment angle estimated by the OBA are shown in Figure 13 and Figure 14. The figures shows that the proposed method can correctly estimate the OD scale factor error, and the SINS installation angle error can be estimated after the vehicle turning as we expected. The estimate results of attitude error are shown in Figure 15. As can be seen from Figure 15, the heading error can reach an accuracy of 5′ within 200 s, and the two-level misalignment angles can reach an accuracy of 1′.

## 5. Conclusions

This paper has proposed a novel algorithm for the joint estimation of SINS/odometer attitude and associated parameters including the odometer scale factor, lever arm, IMU misalignment angle and inertial sensor biases. The global observability analysis of INS/odometer system is conducted at first. Then, based on the observability analysis results, an integration algorithm for identifying odometer scale factor and IMU misalignment angle was designed, and the initial alignment and calibration algorithm based on optimal algorithm is established. Later on, the initial alignment and calibration problem is posed as a unit quaternion-constrained optimization on attitude, lever arm, accelerometer bias and gyroscope drift, and the Newton-Lagrange algorithm is derived to solve the problem. Finally, simulation and experiment studies show that this new technique has the capability of self-initialization and calibration without any prior attitude and sensor noise information, and the performance of OBA method is superior to the EKF method during the angular motion.

## Figures and Tables

**Table 1:** The estimate results of typical motion simulation.

Estimate Error	EKF	OBA
Attitude (min)	[0.0032 0.0042 0.0949]^T^	[−0.0023 −0.0023 0.0413]^T^
Lever arm (m)	[−0.0071 0.0347 −0.0387]^T^	[0.0010 0.0037 −0.0032]^T^

**Table 2:** The estimate results of angular motion simulation.

Estimate Error	EKF	OBA
Attitude (min)	[0.0134 0.0107 −0.2233]^T^	[−0.0130 −0.0087 0.0878]^T^
Lever arm (m)	[−0.0242 0.0308 0.0028]^T^	[−0.0033 0.0005 −0.0004]^T^
